# Association between Blood Viscosity and Cardiovascular Risk Factors in Patients with Arterial Hypertension in a High Altitude Setting

**DOI:** 10.7759/cureus.3925

**Published:** 2019-01-21

**Authors:** Erika D Taco-Vasquez, Francisco Barrera, Marcos Serrano-Duenas, Enoe Jimenez, Alexander Rocuts, Efrain Riveros Perez

**Affiliations:** 1 Anesthesiology, Cleveland Clinic, Cleveland, USA; 2 Internal Medicine, Pontifical Catholic University of Ecuador, Quito, ECU; 3 Anesthesiology, The Medical College of Georgia, Augusta University, Augusta, USA

**Keywords:** viscosity, whole blood viscosity, cardiovascular risk factors, arterial hypertension

## Abstract

Background

Blood viscosity is a determinant of vascular resistance, and it is expected to contribute to blood pressure. Arterial hypertension (HTN), in addition to other cardiovascular risk factors, contribute to cardiac morbidity. Our study aimed to establish the association between cardiovascular risk factors including HTN and whole blood viscosity in Ecuadorian patients.

Material and methods

We studied 132 patients with the diagnosis of HTN. Fifteen cardiovascular risk factors were analyzed. The association between whole blood viscosity (WBV) and the number of cardiovascular risk factors was studied. The association between blood viscosity and risk factors was analyzed.

Results

One hundred and thirty-two patients were analyzed. Blood viscosity was associated with a number of cardiovascular risk factors. Creatinine, uric acid, total cholesterol, and low-density lipoprotein (LDL) values were significantly higher in patients with high blood viscosity.

Conclusion

Blood viscosity is a physiological variable associated with a number of cardiovascular risk factors in hypertensive patients. Such risk factors are related to renal function and lipid profiles. In high-altitude residents, polycythemia is common, and the consequences of high hematocrit on cardiovascular morbidity in this setting deserve special attention and warrant further research.

## Introduction

Whole blood behaves as a non-Newtonian fluid, whose viscosity depends on shear rate. At low shear rates, blood cells aggregate, increasing viscosity, whereas the opposite happens at a high shear rate [1-3]. Increased whole blood viscosity (WBV) is associated with higher morbidity and mortality in some settings, including cardiovascular and cerebrovascular disease [2]. Blood viscosity is directly proportional to endothelial shear stress, which in turn is determined by vessel diameter, through the release of endothelial vasoactive factors [4-5]. Therefore, vasodilation increases shear stress and fluid viscosity without changing the flow rate [6]. In conditions associated with elevated levels of WBV, endothelial shear stress is further altered [5].

In the human microvasculature, shear stress induces nitric oxide-mediated vasodilation. This phenomenon is blunted in hypertensive patients. By contrast, small vessels of patients with hypercholesterolemia exhibit normal vasodilation induced by shear stress despite diminished nitric oxide activity [7]. Atheroma formation involves the buildup by the accumulation of low-density lipoprotein in relation to elevated blood viscosity in areas of low flow, thus predisposing to thrombosis [8-9]. High-density lipoprotein, on the other hand, is protective against atherosclerosis by decreasing blood viscosity in areas that are prone to thrombus formation. Additionally, blood viscosity contributes to the development of HTN by increasing systemic vascular resistance [9]. The combination of HTN and high WBV has been identified as a contributor to cardiovascular complications including left ventricular hypertrophy [10].

In Ecuador, the morbidity rates for HTN in 2007 and 2011 were 6.20 and 6.83, respectively [11]. HTN by itself increases the risk of adverse cardiac events two to three times [12]. Due to its high prevalence, it is expected that HTN will contribute to approximately 35% of the overall cardiovascular risk and atherosclerosis. Several studies reveal the association between HTN and metabolic conditions such as hypercholesterolemia, hypertriglyceridemia, low high-density lipoprotein (HDL), obesity, and diabetes mellitus [12]. Our study was aimed at establishing the association between the cardiovascular risk factors including HTN and WBV in Ecuadorian patients.

## Materials and methods

After approval by the Ethics Committee of Pontificia Universidad Catolica del Ecuador, we studied a population of 180 patients with the diagnosis of HTN based on the criteria outlined in the Seventh Report of the Joint National Committee on Prevention, Detection, Evaluation, and Treatment of High Blood Pressure [12]. We retrospectively evaluated blood viscosity of patients presenting to the internal medicine outpatient clinic, with age range between 40 and 90 years and diagnosis of HTN, who had blood cell count and protein levels ordered for a clinical indication. Patients presenting with heart failure with an ejection fraction of less than 40% as well as those with a diagnosis of chronic obstructive pulmonary disease or obstructive pattern on pulmonary function tests were excluded. Demographic information included age, sex, ethnicity, family history of arterial hypertension, body mass index (BMI), and smoking history. Noninvasive blood pressure, blood cell counts, total protein levels, total cholesterol, HDL, low-density lipoprotein (LDL), and triglycerides were recorded. Creatinine clearance based on weight, age, and gender was calculated for each patient. Blood viscosity was calculated from hematocrit and total protein values using the equation: WBV = 0.12 × HCT + 0.17 (TP–2.07), where HCT denotes hematocrit (%) and TP the total serum protein level (g/L) [3]. The formula estimates the blood viscosity at a level of shear force cut at 208 s^-1^.

We assigned records to one of two groups: group 1 included patients with increased WBV, and group 2 had normal WBV. We defined high WBV as being equal or higher than 19.02 centipoise. The association between WBV and the number of cardiovascular risk factors was analyzed using Student’s *t*-test and Chi-square test for the different types of variables. A calculated *p*-value less than 0.05 was considered statistically significant. We also compared the WBW net values according to the number of cardiovascular risk factors present in each patient, through the statistical contrast of Kruskall-Wallis. All statistical analyses were performed with SPSS 20.

## Results

One hundred and eighty patients met the inclusion criteria. One hundred and thirty-two patients had all the information needed for analysis. Eighty-three percent of patients were male. Table 1 shows means and standard deviations for demographic and clinical variables. Fourteen percent (*n *= 18) of patients were found to have elevated blood viscosity as determined by the WBV equation described in the Methods section [3].

**Table 1:** Clinical and demographic characteristics in patients with arterial hypertension

Variable	Mean (SD)
Age (years)	62 (12)
BMI (kg/m^2^)	29 (5)
Age of diagnosis (years)	53 (12)
Time of disease (years)	10 (9)
SBP (mmHg)	116 (10)
DBP (mmHg)	70 (5)
Creatinine (mg/dL)	1,1 (0,3)
Uric acid (mg/dL)	5,8 (1)
Total cholesterol (mg/dL)	221 (51)
HDL (mg/dL)	61 (14)
LDL (mg/dL)	121 (43)
TG (mg/dL)	192 (89)
Cl Cr (ml/min/1,73m^2^)	57 (20)
Hematocrit (%)	45.8 (3.3)
Total proteins (g/dL)	7.5 (0.5)
Blood viscosity	17.9 (1.04)

**Figure 1:**
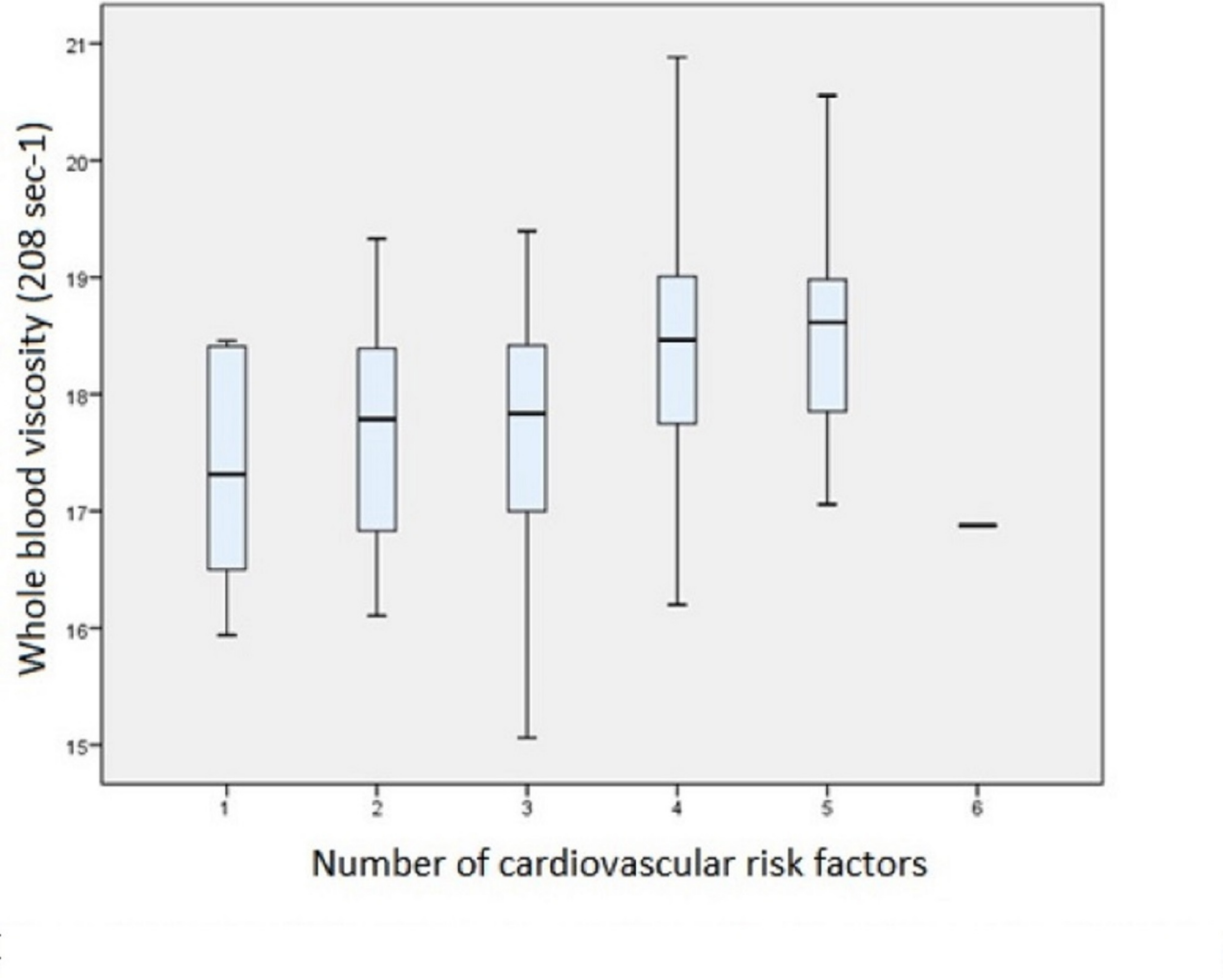
Box plot of blood viscosity and the number of cardiovascular risk factors

**Figure 2:**
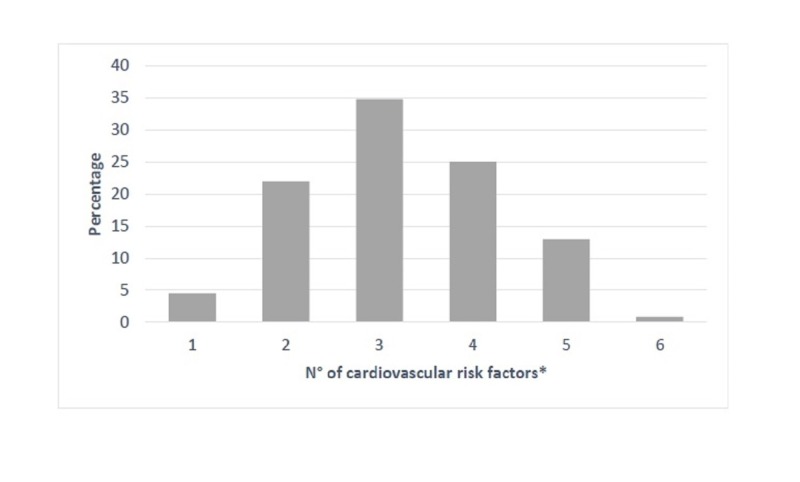
Percentage of cardiovascular risk factors in hypertensive patients

We analyzed six cardiovascular risk factors (RF): age, family history, smoking, dyslipidemia, obesity, and kidney disease. We scored the average number of RF for each patient. In our sample, 5% of patients (*n* = 6) had only one RFs, 22% (*n* = 29) had two RFs, 35% (*n* = 46) had three RFs, 25% (*n* = 33) had four RFs, 13% (*n* = 17) had 5 RFs, and 1% (*n* = 1) had 6 RFs (Figure 1). Blood viscosity was associated with the number of cardiovascular RFs (*p* = 0.001; Table 2 and Figure 2).

**Table 2:** Descriptive statistical data of blood viscosity according to the number of risk factors

N° of CVRF	1	2	3	4	5	6	Chi^2^	P
n	6	29	46	33	17	1		
Mean BV	17.3	17.6	17.7	18.4	18.5	16.9	19.6	0,001*

The observed increases in blood viscosity in association with the number of RFs did not show any relationship of dependence. On the other hand, creatinine, uric acid, total cholesterol, and LDL levels were associated with increased viscosity, whereas creatinine clearance decreased with increased values of viscosity. The means and *p* values of the variables are shown in Table 3.

**Table 3:** Cardiovascular risk factors in relation to blood viscosity

Risk factor	Normal WBV	High WBV	t		p
Age (years)	63	65	0.67	0.507	
BMI (kg/m^2^)	29.4	31.1	1.48	0.155	
Age of diagnosis (years)	54	52	0.65	0.518	
Time of disease (years)	9.7	13.8	1.91	0.059	
SBP (mmHg)	116	119	1.18	0.24	
DBP (mmHg)	69	71	1.11	0.27	
Creatinine (mg/dL)	1.1	1.4	3.13	0.002*	
Uric acid (mg/dL)	5.7	6.8	2.15	0.033*	
Total cholesterol (mg/dL)	217	250	2.64	0.009*	
HDL (mg/dL)	61	65	0.94	0.351	
LDL mg/dL)	117	144	2.5	0.014	
TG (mg/dL)	191	203	0.53	0.6	
Cl Cr (ml/min/1,73m^2^)	58.9	47.5	2.29	0.023*	

Age, sex, age at diagnosis, years of illness, family history, smoking status, body mass index, systolic and diastolic pressures, HDL cholesterol, triglyceride levels, and microalbuminuria did not show an association with blood viscosity values.

## Discussion

Our study found a correlation between WBV and the number of cardiovascular risk factors [13-15]. The study also found that creatinine, uric acid, total, and LDL cholesterol levels have a positive association with WBV values. The pathophysiology of atherosclerosis is a complex multifactorial process. Blood viscosity contributes to the development of atherosclerosis in virtue of its ability to increase blood flow-related shear stress [16]. Laminar shear stress is known to be crucial for normal vascular functioning, by regulating vascular caliber and inhibiting endothelial cell proliferation, thrombosis, and inflammation. Thus, shear stress is atheroprotective. Our results are consistent with the effect of blood viscosity and vascular shear stress with atheroma formation [17]. However, since we cannot establish a causal relationship between lipid profiles and blood viscosity, it is still possible that the changes in blood viscosity are the consequence rather than the cause of changes in lipid profile [18]. At this point, we can affirm that hyperlipidemia coexists with increased blood viscosity. It is recognized that flow disturbance occurring near arterial bifurcations, branch ostia, and curvatures is associated with atheroma formation. Non-laminar flow promotes changes to endothelial gene expression, cytoskeletal arrangement, wound repair, leukocyte adhesion as well as to the vasoreactive, oxidative, and inflammatory states of the artery wall [19]. Shear stress is critically important in the regulation of atheroprotective mechanisms of vascular beds [17]. We believe that the effect of blood viscosity on shear stress is in part responsible for the formation of atherosclerotic plaques in areas of vascular non-laminar flow, and intervention of this variable might have a clinical impact on vascular health [20].

Mehmet et al. demonstrated that blood hyperviscosity is an independent risk factor for impaired coronary microvascular perfusion and angiogenesis [13]. In the Edinburg study, the relationship between WBV and adverse cardiovascular events (ischemic heart disease and stroke) was examined in 1,592 people. A cumulative incidence was 17.1% of fatal and non-fatal events occurred after five years of follow-up. After adjustment for age and sex, blood viscosity (3.70 v 3.55 mPa.s), hematocrit (46.2 v 45.7%), hematocrit-corrected blood viscosity (3.57 v 3.48 mPa.s), plasma viscosity (1.35 v 1.33 mPa.s), and fibrinogen level (2.88 v 2.67 g/l) were significantly higher in subjects who experienced events than in subjects who did as those of conventional risk factors (smoking habit, diastolic blood pressure, and levels of LDL cholesterol). After adjustment for these conventional risk factors, the associations of blood viscosity and hematocrit remained significant for stroke, but not for total events, whereas those of plasma viscosity and fibrinogen remained significant for both total events and for stroke.

Chronic kidney disease (CKD) is a significant risk factor for the development of cardiovascular disease and a major contributor to cardiac-related mortality. On the other hand, blood viscosity has been associated with decreased renal function and increased urinary excretion of albumin in hypertensive patients without renal disease [10]. In a study in patients with mitral annular calcification and newly diagnosed CKD, the authors found that patients with values of WBV higher than 5.2 cP had lower survival rates compared to patients with WBV values below that level [16]. Our study showed a correlation between WBV and creatinine levels. Gordge et al. found that WBV is associated with progression of renal impairment in patients with diabetes mellitus [21]. Although a causality relationship cannot be clearly established in the context of kidney disease, the intervention of the viscosity factor might prove useful to avoid complications related to vascular stasis such as dialysis access patency and vascular disease [22].

Hypertensive patients in our study exhibited a high prevalence of lipid profile abnormalities when blood pressure was poorly controlled. Gebrie et al. showed that red blood cell counts increase in parallel with the severity of HTN [18]. Traditionally recognized cardiovascular risk factors (e.g., triglycerides, obesity, and cholesterol levels) have been found to be positively related to WBV or plasma viscosity [9]. In our study, only LDL and total cholesterol were associated with WBV levels. This finding could be attributed to the sample size used in this study; however, our findings might be pointing to the possibility that the WBV has a more pronounced effect on total and LDL cholesterol compared to other lipids, opening the potential for intervention of blood viscosity as a strategy to affect these two types of cholesterol.

Blood viscosity is elevated in conditions of decreased alveolar pressure of oxygen, as happens in high altitude cities. This study was conducted in Quito, Ecuador (2800 m. of altitude), where the atmospheric pressure level is lower compared to sea level (526 mm Hg). Although it would be expected that high hematocrit secondary to altitude results in increased endothelial shear stress, individuals living at high altitude exhibit increases in red cell deformability and endothelial production of nitrous oxide [23-24]. Additionally, shear stress promotes synthesis of endothelial local vasodilators. This may explain the low mortality from ischemic cardiac and cerebral disease in high altitude residents [25].

Our study has limitations. The retrospective nature of the data collection allowed us to draw conclusions regarding the association of blood viscosity and cardiovascular risk factors; however, since causality could not be determined, hyperviscosity could be just an epiphenomenon in the complex network of pathophysiological mechanisms underlying vascular disease. The lack of association of blood viscosity with systolic and diastolic blood pressure seems to be counterintuitive and could be related to the small sample size. We recommend that future research efforts focus on the conduction of randomized trials to elucidate the effect of interventions on blood viscosity to affect individual cardiovascular risk factors.

## Conclusions

Our results show that WBV is a physiological variable associated with a number of cardiovascular risk factors in hypertensive patients. Such risk factors are related to renal function and lipid profiles. In high-altitude residents, polycythemia is common, and the consequences of high hematocrit on cardiovascular morbidity in this setting deserve special attention and warrant further research.
